# Influence of silica–alumina support ratio on H_2_ production and catalyst carbon deposition from the Ni-catalytic pyrolysis/reforming of waste tyres

**DOI:** 10.1177/0734242X17722207

**Published:** 2017-08-08

**Authors:** Yeshui Zhang, Yongwen Tao, Jun Huang, Paul Williams

**Affiliations:** 1 School of Chemical & Process Engineering, University of Leeds, Leeds, UK; 2 Australian Institute for Nanoscale Science and Technology, School of Chemical & Biomolecular Engineering, The University of Sydney, Sydney, Australia

**Keywords:** Waste, tyres, pyrolysis, catalyst, hydrogen, carbon nanotubes

## Abstract

The influence of catalyst support alumina–silica in terms of different Al_2_O_3_ to SiO_2_ mole ratios containing 20 wt.% Ni on the production of hydrogen and catalyst coke formation from the pyrolysis-catalysis of waste tyres is reported. A two-stage reactor system was used with pyrolysis of the tyres followed by catalytic reaction. There was only a small difference in the total gas yield and hydrogen yield by changing the Al_2_O_3_ to SiO_2_ mole ratios in the Ni-Al_2_O_3_/SiO_2_ catalyst. The 1:1 ratio of Al_2_O_3_:SiO_2_ ratio produced the highest gas yield of 27.3 wt.% and a hydrogen production of 14.0 mmol g^-1^_tyre_. Catalyst coke formation decreased from 19.0 to 13.0 wt.% as the Al_2_O_3_:SiO_2_ ratio was changed from 1:1 to 2:1, with more than 95% of the coke being filamentous-type carbon, a large proportion of which was multi-walled carbon nanotubes. Further experiments introduced steam to the second-stage reactor to investigate hydrogen production for the pyrolysis-catalytic steam reforming of the waste tyres using the 1:1 Al_2_O_3_/SiO_2_ nickel catalyst. The introduction of steam produced a marked increase in total gas yield from ~27 wt. % to ~58 wt.%; in addition, hydrogen production was increased to 34.5 mmol g^-1^ and there was a reduction in catalyst coke formation to 4.6 wt.%.

## Introduction

Waste tyre disposal represents a major worldwide problem. In 2014, 4.67 million tonnes of tyres were produced in Europe (ETRMA, 2015), eventually leading to a similar number of waste tyres arising annually to be managed. The current main treatment routes for waste tyres include fuel for cement kilns as a replacement for fossil fuels, recycling as rubber crumb for sports fields or playgrounds, retreading of part worn tyres, civil engineering applications and export (Williams, 2013). However, recently, there has been much interest in advanced thermal treatment technologies such as pyrolysis and gasification as a means of producing higher-value products from waste tyres. For example, the solid char product from pyrolysis of the tyres can be upgraded to produce high surface area activated carbons (Cunliffe and Williams, 1998; Nunes et al., 2011; Zabaniotou et al., 2004). In addition, the product oil and gas can be upgraded for the production of high-grade liquid fuels, hydrogen or aromatic chemicals (Leung and Wang, 2003; Williams, 2013; Williams and Brindle, 2003; Xiao et al., 2008; Zhang et al., 2015).

The production of hydrogen from waste tyres has been investigated by only a few research groups (Elbaba and Williams, 2012; Lerner et al., 2012). However, there is predicted to be high demand for hydrogen in the future to support the proposed hydrogen economy. Whilst hydrogen is currently mostly produced from fossil fuels, production of waste materials such as waste tyres would represent a novel route to recycle waste tyres. Alternative feedstocks for hydrogen production have included biomass, waste plastics and municipal solid waste (Wang et al., 2012; Wu and Williams, 2009a).

Catalysts are extensively used for increasing hydrogen yield from the steam reforming of hydrocarbon sources (Sutton et al., 2001; Wu and Williams, 2009a). Catalysts containing metals such as Ru, Pt and Rh have been shown to be effective for hydrogen production; however, nickel is a preferred metal due to its low cost (Sehested, 2006; Srinakruang et al., 2005). The type of support used to support the nickel will also influence the catalytic activity of the catalyst through the characteristics of surface area, porous structure and mechanical strength, and chemical interaction between the metal and catalyst. For example, Miyazawa et al. (2006) investigated the performance of nickel-based catalysts on various supports for the steam reforming of tars from biomass pyrolysis. The catalyst supports tested were Al_2_O_3_, ZrO_2_, TiO_2_, CeO_2_ and Ni/MgO. It was reported that the production of hydrogen was influenced by the support material, with Ni/Al_2_O_3_, Ni/ZrO_2_ and Ni/TiO_2_ catalysts producing the largest hydrogen yields. The interaction between the nickel and the support was shown to influence the metal dispersion and the size of the metal particles, which in turn affected catalyst activity. Wu and Williams (2009a) reported on the use of Ni/Al_2_O_3_, Ni/MgO, Ni/CeO_2_ and Ni/ZSM-5 catalysts with 10 wt.% nickel for the pyrolysis-catalytic steam reforming of waste plastics. The Ni/MgO catalyst produced the lowest yield of hydrogen and the Ni/ZSM-5 catalyst the highest yield, which could be linked with the surface area of the catalysts. In addition, Ni/Al_2_O_3_ and Ni/CeO_2_ catalysts also showed significant amounts of carbon deposition. Inaba et al. (2006) investigated Ni/SiO_2_, Ni/ZrO_2_, Ni/CeO_2_ and a series of zeolites for use as catalysts in hydrogen production from the gasification of cellulose. The highest hydrogen yield was with the Ni/SiO_2_ catalyst. Srinakruang et al. (2005) investigated the gasification of tar using nickel catalysts supported on SiO_2_–Al_2_O_3_, Al_2_O_3_ and dolomite. The nickel catalysts on SiO_2_–Al_2_O_3_ and Al_2_O_3_ produced larger amounts of carbon deposition than the Ni-dolomite catalyst, which led to deactivation.

Alumina (Al_2_O_3_) is the most commonly used catalyst support for nickel for investigations of hydrogen production because of its effectiveness in hydrogen production and its chemical and physical stability and mechanical strength (Simell et al., 1997). However, the deactivation of the Ni/Al_2_O_3_ catalyst by carbon (coke) deposition and sintering are problematic (Sehested, 2006). Nickel catalysts with a SiO_2_ support have also shown to be effective for hydrogen production (Blanco et al., 2014; Inaba et al., 2006; Sutton et al., 2001) and are also low cost. Mixed alumina–silica supports would generate surface acid groups, which could enhance the activity of supported Ni catalysts due to the bifunctional property. It is therefore interesting to compare Ni-based catalysts with different Al_2_O_3_/SiO_2_ ratios for their effectiveness as a nickel catalyst support for hydrogen production and also in terms of detrimental catalyst coke deposition. Using waste tyres as feedstock for hydrogen production also extends the knowledge in relation to this lesser researched feedstock for hydrogen production.

In this paper, the effect of the Al_2_O_3_ to SiO_2_ ratio in Ni-based catalysts for the production of hydrogen from waste tyres has been investigated. Ni/Al_2_O_3_/SiO_2_ catalysts containing 20 wt.% nickel with four different Al_2_O_3_ to SiO_2_ mole ratios of, 3:5, 1:1, 3:2 and 2:1 were investigated via pyrolysis-catalysis and pyrolysis-catalytic steam reforming of the waste tyre. The influence of different water injection rates introducing steam to the process was also examined to determine the effect on hydrogen production and catalyst coke deposition.

## Material and methods

### Materials

The waste tyre sample was prepared from shredded truck tyres by removing the metal and cutting into particles of ∼6 mm. A Carlo Erba Flash EA1112 elemental analyser was used to determine the elemental composition, which was 81.2 wt.% carbon, 7.2 wt.% hydrogen, 0.8 wt.% nitrogen, and 2.1 wt.% sulphur (Zhang et al., 2015).

The catalysts used in the experiments were 20 wt.% Ni/Al_2_O_3_/SiO_2_ catalysts with four different Al_2_O_3_ to SiO_2_ mole ratios (3:5, 1:1, 3:2, 2:1). The catalysts were synthesized by an incipient wetness method, where 20 wt.% of Ni was impregnated onto the alumina–silica mixture support that was prepared by a sol-gel method. Silica and aluminium isopropoxide were purchased from Sigma-Aldrich and were the precursors for the silica and alumina. The calculated amounts of SiO_2_ powder and aluminium isopropoxide powder were mixed with distilled water, followed by filtering the solution with distilled water. The obtained Al(OH)_3_-SiO_2_ mixtures were aged in air overnight and kept in an oven at 40°C for 1 day. Finally, the dry solids were ground into fine powder as catalyst support for the impregnation preparation step. The 20 wt.% nickel was impregnated on the Al_2_O_3_ and SiO_2_ matrix support via dissolution of the calculated amount of nickel nitrate in ethanol. The Al_2_O_3_ and SiO_2_ catalyst support was then added with continuous stirring until the mixture became a slurry. The impregnation process ended with drying the slurry in an oven overnight to evaporate all of the moisture. The last step was to calcine the dry solids at 750°C in an air atmosphere with a heating rate of 2°C min^-1^ and holding time of 3 hours.

### Experimental system

**Figure 1.**
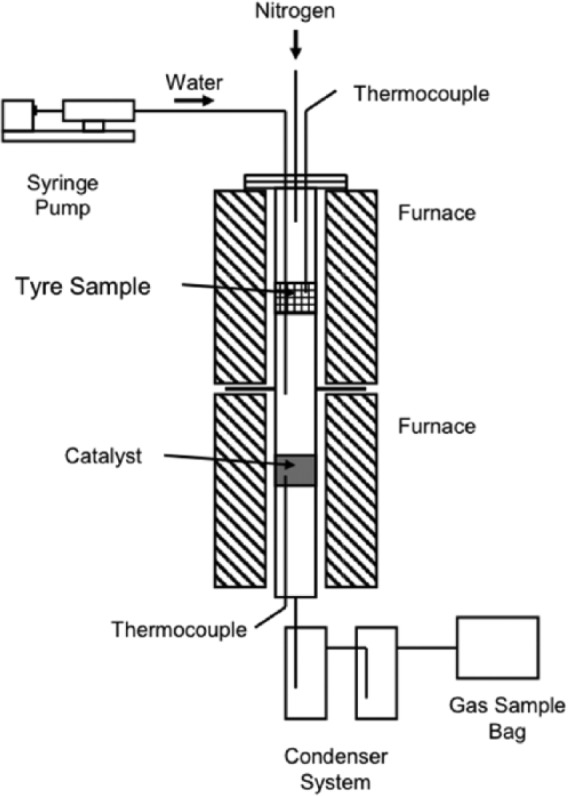
Schematic diagram of the two-stage fixed bed reactor system.

The reactor for the pyrolysis-reforming of waste tyres consisted of a two-stage fixed bed reactor. Figure 1 shows the schematic diagram of the experimental system. After being pyrolysed in the first pyrolysis stage, the gas products from the tyres are passed directly to the second stage where either catalysis or catalytic reforming takes place (Elbaba and Williams, 2013; Elbaba et al., 2010; Zhang et al., 2015).

The reactors for both stages were constructed of stainless steel with a diameter of 2.2 cm and a height of 16 cm. The two stages are heated separately by two external electrical heaters, which controlled the heating rate and the target temperature for the experiments. Both of the stages were purged with nitrogen gas continually. The real-time internal temperatures of the reactors were monitored by thermocouples near the catalyst and tyre sample (Elbaba and Williams, 2012).

The reforming and pyrolysis temperature was 800 and 600°C, respectively. In each of the experiments, 1.0 g tyre sample and 0.5 g catalyst were introduced. The heating rate of the pyrolysis stage was 10°C min^-1^ to the final temperature of 600°C, and was held at that temperature for 20 minutes. No steam was introduced to the experiments with different SiO_2_:Al_2_O_3_ ratios catalysts. Experiments with steam reforming were performed with different water injection rates where experiments were carried out with the catalyst which produced the highest hydrogen yield.

### Analytical methods

X-ray diffraction was used to identify the composition of the freshly prepared catalysts. The gaseous products from the pyrolysis-reforming of the waste tyres were collected by a Tedlar™ gas sample bag, and further analysed by packed column gas chromatography (GC). Permanent gases which included H_2_, CO, O_2_, N_2_ and CO_2_ were analysed by a Varian 3380 GC/TCD with a 2 m long, 2 mm diameter and 60–80 mm mesh molecular sieve packed column. Hydrocarbons including C_1_–C_4_ were analysed by a Varian 3380 with a flame ionization detector with 80–100 mesh HayeSep column and nitrogen carrier gas. The gases are reported on a nitrogen-free basis.

Thermogravimetric analyser-temperature programmed oxidation (TGA-TPO) was used to analyse the reacted catalysts to investigate the types of carbon formed during the pyrolysis-catalytic/reforming of waste tyres. Raman spectroscopy was used to determine the degree of graphitization of the carbon productions. The results were obtained using a Renshaw Invia Raman spectrometer at a wavelength of 514 nm and Raman shifts between 100 and 3000 cm^-1^. Scanning electron microscopy (SEM) and transmission microscopy (TEM) were used to observe the surface characteristics of the fresh and reacted catalysts to help determine the structure of the carbon deposition on the catalyst surface. The instruments used were a Hitachi SU8230 SEM and a FEI Tecnai TF20 TEM.

## Results and discussion

### Product yields and gas composition

#### Influence of different Al_2_O_3_ to SiO_2_ ratios for the pyrolysis-catalysis of waste tyres

Table 1 shows the product yield from pyrolysis-catalysis of waste tyres with different 20 wt.% Ni/Al_2_O_3_/SiO_2_ catalysts in relation to different Al_2_O_3_ to SiO_2_ ratios. The solid residue left after pyrolysis, carbon on the catalyst and oil were measured by the weight difference before and after reaction. The gas yield was calculated by comparing the concentration of product gases and reference gas (nitrogen). The results produced a mass balance of between 94 and 95 wt.%, where errors could occur from condensation of heavy molecular weight hydrocarbons within the reactor, or variations in the flow rate of nitrogen carrier gas, etc.

**Table 1.** Product yields and gas concentrations from the pyrolysis-catalysis of waste tyres with a 20 wt.% Ni/Al_2_O_3_/SiO_2_ catalyst with different Al_2_O_3_ to SiO_2_ ratios (3:5, 1:1, 3:2 and 2:1).

Al_2_O_3_:SiO_2_ ratio	3:5	1:1	3:2	2:1
Gas yield (wt.%)	23.3	27.3	25.9	25.3
Liquid yield (wt.%)	14.0	13.0	15.0	17.0
Residue (wt.%)	39.0	36.0	39.0	39.0
Catalyst coke (wt.%)	18.0	19.0	14.0	13.0
Hydrogen production (mmol g^-1^_tyre_)	11.5	14.0	12.0	12.0
CO production (mmol g^-1^_tyre_)	2.4	3.3	2.6	2.3
Syngas production (mmol g^-1^_tyre_)	13.9	17.3	14.6	14.3
Gas concentrations (vol. %)				
CO	11.6	13.3	11.9	10.7
H_2_	55.1	56.1	54.9	55.0
CH_4_	23.3	22.0	21.4	20.9
CO_2_	0.0	0.0	0.0	0.0
C_2_–C_4_	4.8	4.5	6.0	4.6
Calorific value (MJ m^-3^)	20.0	19.5	20.7	18.7

From Table 1, the results show that as the Al_2_O_3_ to SiO_2_ ratio was increased, the influence on hydrogen production was small, with the highest amount of 14.0 mmol g^-1^ hydrogen produced at the Al_2_O_3_:SiO_2_ ratio of 1:1. The calorific values of the produced gases did not change significantly by changing the Al_2_O_3_ to SiO_2_ ratios, which were in the range of 18.7 to 20.7 MJ m^-3^. There was no significant difference in liquid yield between the different catalysts with different Al_2_O_3_ to SiO_2_ ratios. It can be concluded that the catalyst with an Al_2_O_3_:SiO_2_ ratio 1:1 showed the best performance in terms of the gas yield and hydrogen production. Table 1 shows that the increased content of Al_2_O_3_ in the 20 wt.% Ni/Al_2_O_3_/SiO_2_ catalyst at the higher Al_2_O_3_ to SiO_2_ ratio produced reduced amounts of catalyst carbon deposition, decreasing from ~18/19 to ~13 wt.%.

#### Influence of different water injection rates

The 20 wt.% nickel catalyst at a Al_2_O_3_ to SiO_2_ ratio of 1:1 produced the highest yield of hydrogen in the pyrolysis-catalysis experiments. Therefore, further work was carried out to increase the production of hydrogen from the waste tyres by introducing steam (injected water) to the second stage to produce a pyrolysis-catalytic steam reforming process. Table 2 shows the product yields and gas compositions from waste tyre pyrolysis-catalytic steam reforming with the 20 wt.% Ni/Al_2_O_3_/SiO_2_ (1:1) catalyst at different water injection rates. The gas yields with water injection rate at 2 and 5 ml h^-1^ were similar at 58.0 and 57.8 wt.%, respectively, which were much higher than the experiments without water at only 27.3 wt.%. The hydrogen production from the process showed a similar trend, at 14.0, 33.8 and 34.5 mmol g^-1^ for the water injection rates of 0, 2 and 5 ml h^-1^, respectively.

**Table 2.** Product yields and gas concentrations from the pyrolysis-steam reforming of waste tyres with a 20 wt.% Ni/Al_2_O_3_/SiO_2_ catalyst with an Al_2_O_3_:SiO_2_ ratio of 1:1 at different water injection rates (No water, 2 and 5 ml h^-1^).

Tyre + 20%Ni/Al_2_O_3_/SiO_2_			
In relation to sample & reacted water	No water	2ml h^-1^	5ml h^-1^
Gas yield (wt.%)	27.3	58.0	57.8
Oil yield (wt.%)	13.0	2.7	7.0
Residue (wt.%)	36.0	26.3	24.6
Catalyst coke (wt.%)	19.0	10.1	4.6
Mass balance (wt.%)	95.3	97.2	94.0
Hydrogen production (mmol g^-1^_tyre_)	14.0	33.8	34.5
CO production (mmol g^-1^_tyre_)	3.3	11.41	10.48
Syngas production (mmol g^-1^_tyre_)	17.3	45.22	45.00
Gas concentrations (vol. %)			
CO	13.26	19.11	17.41
H_2_	56.06	56.65	57.33
CH_4_	22.01	8.43	8.52
CO_2_	0.00	12.80	14.58
C_2_–C_4_	4.47	1.55	1.75
Reacted water (g)	None	0.48	0.50
In relation to sample only			
Gas yield (wt.%)	27.34	85.90	87.03
Oil yield (wt.%)	13.00	4.04	10.46
Residue (wt.%)	36.00	39.00	37.00
Catalyst coke (wt.%)	19.00	15.00	7.00
Mass balance (wt.%)	95.34	143.94	141.49
Calorific value (MJ m^-3^)	19.47	13.31	10.72

These increases in total gas yield and hydrogen production are attributed to the reacted water, since at a water injection rate of 5 ml h^-1^ the reacted water was 0.5 g, whereas at 2 ml h^-1^ the reacted water was 0.48 g. Table 2 also shows the calorific values of the gas product. The results show that as the water injection rate was increased from 0 to 5 ml h^-1^, there was decrease in the calorific value of the product gases, from 19.47 to 10.72 MJ m^-3^, due mainly to the decrease of the methane and C_2_–C_4_ hydrocarbons due to the catalytic steam reforming process of these hydrocarbons.

Table 2 shows that the product oil and carbon deposition on the catalyst decreased as the amount of water injected into the second stage reforming process was increased. Wu and Williams (2009b) reported that increasing water flow rate contributes to a significant decrease of carbon deposition on the catalyst in their experiments in relation to hydrogen production from the pyrolysis-catalytic reforming of polypropylene. The results show the oil yields were 13.0, 2.7 and 7.0 wt.% at water injection rates of 0, 2 and 5 ml h^-1^, respectively. It should be noted that Table 2 shows data calculated in relation to the amount of injected water and reacted water, which shows that there was 36.00 wt.% of residue with no water added, with 2 and 5 ml h^-1^ water injections, the pyrolysis residues decreased to 26.34 and 24.59 wt.%. However, determination of the pyrolysis residue in relation to tyre sample weight produced pyrolysis residue data that were very similar 37±2 wt.% (as was also shown in Table 1). Table 2 also shows that the CO concentration increased up to 19.11 and 17.41 vol.% from 13.26 vol.% with the introduction of water injection at the rates of 2 and 5 ml h^-1^. The CO_2_ concentration increased from 0 vol.% to 12.80 and 14.58 vol.% at water injection rates of 2 and 5 ml h^-1^. The increase of water injection rate produced a reduction in CH_4_ and C_2_-C_4_ hydrocarbons through steam reforming reactions. The CH_4_ concentration was 22.01 vol.% without water in the experiment, but decreased to 8.43 and 8.52 vol.% at water injection rates of 2 and 5 ml h^-1^, and C_2_–C_4_ concentrations decreased from 4.47 vol.% at no water addition to 1.55 and 1.75 vol.% at water injection rates of 2 and 5 ml h^-1^, respectively.

Turn et al. (1998) found similar results in their experiments for biomass, where an increase of steam to biomass ratio produced an increase in H_2_ and CO_2_ yields. Further increasing steam to the process to high levels can saturate the catalyst surface and reduce hydrogen production; also, higher water inputs require more energy for steam generation (Wu and Williams, 2009b).

### Characteristics of the produced carbon

#### Influences of different Al_2_O_3_ to SiO_2_ ratios

**Figure 2.**
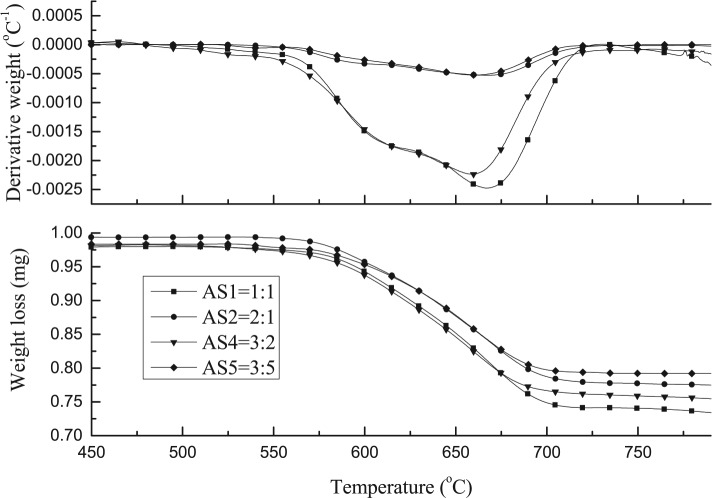
TPO and DTG-TPO results of different used 20 wt.% Ni/Al_2_O_3_/SiO_2_ catalysts with different Al_2_O_3_ to SiO_2_ ratios from pyrolysis-catalysis of waste tyres.

The reacted Ni/Al_2_O_3_/SiO_2_ catalysts with different Al_2_O_3_ to SiO_2_ ratios were further analysed by TGA-TPO. For this analysis the weight loss is mainly due to the oxidation of carbon production on the catalyst surface. Figure 2 shows the TGA-TPO and differential thermogravimetric (DTG)-TPO results of the carbon oxidation for the reacted catalysts in relation to Al_2_O_3_ to SiO_2_ ratio. It can be observed that there are two peaks for all the four different catalysts in the DTG-TPO figure. All of these four DTG-TPO curves show similar trends. There were no peaks below 600°C for any of the catalysts; this could be attributed to the lack of formation of amorphous carbon on the catalyst surface, since such carbons are known to oxidize at lower temperature. Musumeci et al. (2007) have suggested that the amorphous carbons are easily oxidized from the surface of the nickel-based catalyst.

**Figure 3.**
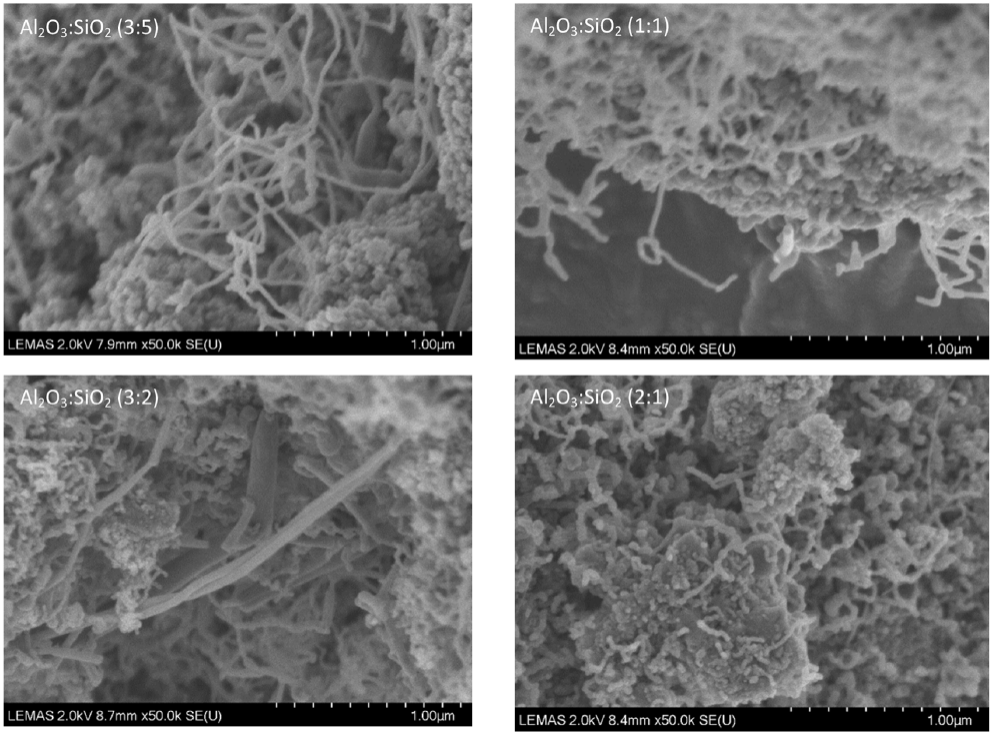
SEM images of carbon deposited on the different reacted 20 wt.% Ni/Al_2_O_3_/SiO_2_ catalysts with different Al_2_O_3_ to SiO_2_ ratios derived from pyrolysis-catalysis of waste tyres.

**Figure 4.**
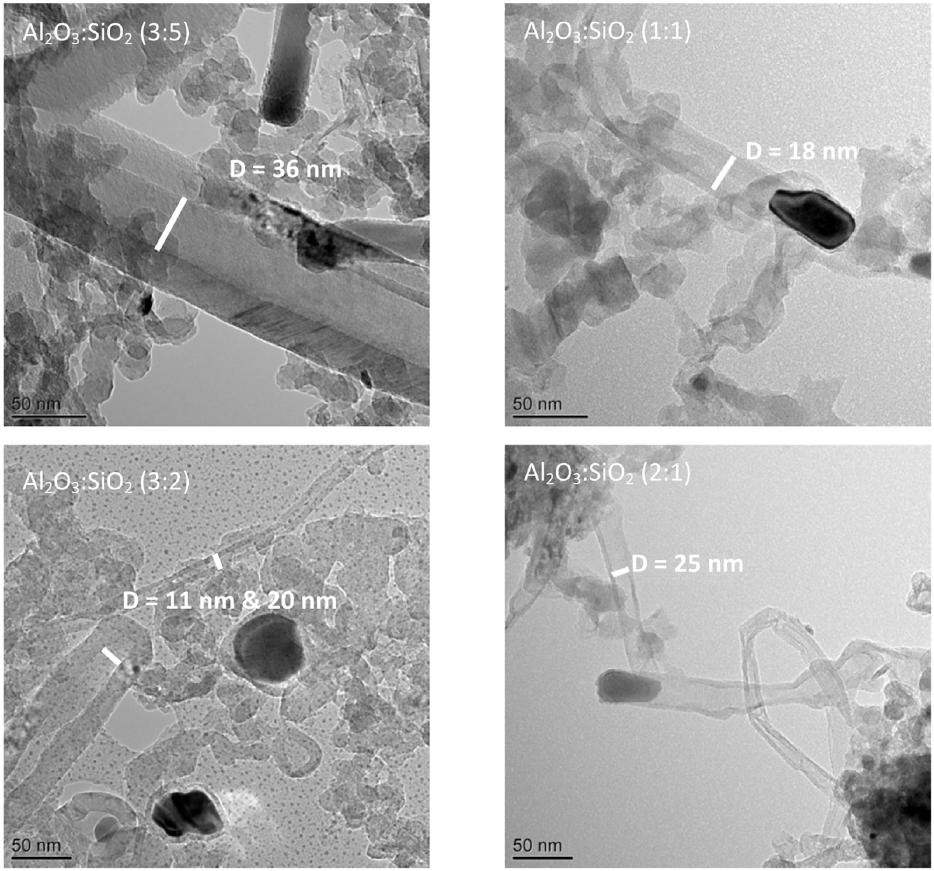
TEM images of carbon deposited on different reacted 20 wt.% Ni/Al_2_O_3_/SiO_2_ catalysts with different Al_2_O_3_ to SiO_2_ ratios derived from pyrolysis-catalysis of waste tyres.

The oxidation peaks for all of the catalysts are present at similar temperatures, in which the first peak occurs at around 600°C and the second peak arises between 650°C and 675°C. The first peak could indicate the oxidization of filamentous carbon which has less graphitization and which is of smaller size. The second oxidization peak could indicate the oxidization of filamentous carbon of larger size and with a higher degree of graphitization. In addition, carbon nanotubes are reported to oxidize at a high temperature above 600°C (Wu and Williams, 2010); Musumeci et al. (2007) also suggested that the more graphitic filamentous carbon are more stable and oxidize at higher temperature with a sharp oxidation peak. Therefore, from the TPO results it can be suggested that the carbon produced in these experiments is mostly filamentous-type carbon with different degrees of graphitization. Examination of the corresponding SEM images, shown in Figure 3, also confirms that the carbon formed on the catalyst surface is mostly filamentous carbon. The corresponding TEM images, shown in Figure 4, also confirm that a large proportion of the filamentous carbon is multi-walled carbon nanotubes (MWCNTs). The first oxidation peaks in the DTG-TPO curves for the Ni catalysts with Al_2_O_3_ to SiO_2_ support ratios of 3:2 and 1:1 are produced at lower temperatures than the other two Al_2_O_3_ to SiO_2_ ratios. This can be assigned to the diameters of the MWCNTs produced with Al_2_O_3_ to SiO_2_ ratios at 3:2 and 1:1 catalysts, which are smaller than the MWCNTs produced with ratios at 3:5 and 2:1 catalyst from the waste tyre pyrolysis-catalysis process. The TEM images in Figure 4 confirm the difference in diameters of the MWCNTs. The inner diameters of the MWCNTs produced with catalysts with Al_2_O_3_ to SiO_2_ ratios at 3:5, 1:1, 3:2 and 2:1 are approximately 36 nm, 18 nm, 11 nm (and 20 nm) and 25 nm, respectively.

Fang et al. (2012) suggested that smaller NiO and CeO_2_ particles of the catalysts contribute to higher reactivity of the smaller size of filamentous carbon. They reported that the oxidization peak of smaller-diameter filamentous carbons in TPO results is larger and shifted to lower temperature compared with the larger-sized filamentous carbon. The largest inner diameter of carbon nanotubes was produced with the highest SiO_2_ content (Al_2_O_3_:SiO_2_ is 3:5) 20 wt.% Ni/Al_2_O_3_/SiO_2_ catalyst. Kukovecz et al. (2000) also found that increasing the amount of silica in the catalyst support slightly increased the thickness of the carbon nanotubes.

Raman spectroscopy analysis was used to determine the degree of graphitization of the carbon deposited on the different catalysts in the pyrolysis-catalysis of waste tyres. The D band, which occurs at the wavelength around 1350 cm^-1^, indicates the presence of amorphous carbons or disordered carbons; the G band, which occurs at the wavelength around 1584 com^-1^, indicates a graphite carbon structure. The ratio of the intensity of the D band to the intensity of the G band (I_D_/I_G_) can help to evaluate the graphitization level of the produced carbons (Zhang et al., 2015). Table 3 shows that the I_D_/I_G_ ratios of catalysts with Al_2_O_3_ to SiO_2_ ratios 3:5, 1:1, 3:2, 2:1 are 0.89, 0.93, 0.91, and 0.93, respectively. The lowest I_D_/I_G_ ratio shows the highest graphitization of the carbon produced on the catalyst at Al_2_O_3_ to SiO_2_ ratio was 3:5, suggesting that the carbons produced have the highest crystallinity.

**Table 3.** Raman analysis of the catalyst coke from the pyrolysis-catalysis of waste tyres with a 20 wt.% Ni/Al_2_O_3_/SiO_2_ catalyst with different Al_2_O_3_ to SiO_2_ ratios and water injection rates.

A_2_O_3_:SiO_2_ ratio and water injection rate	D(y)	G(y)	I_D_/I_G_(y/y)
3:5 without water	4160	4680	0.89
1:1 without water	2628	2819	0.93
3:2 without water	3426	3775	0.91
2:1 without water	7212	7729	0.93
1:1 with 2ml h^-1^ water	12831	14774	0.87
1:1 with 5ml h^-1^ water	6713	7467	0.90

**Figure 5.**
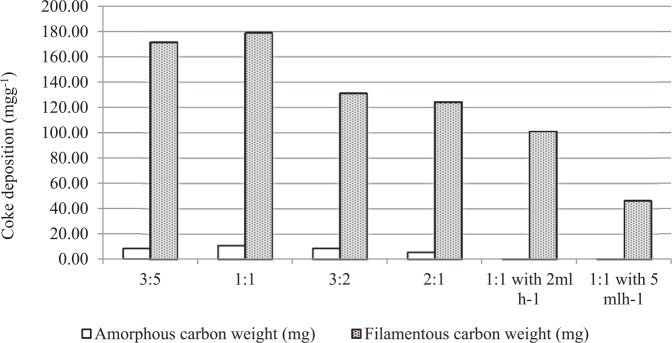
Proportion of amorphous and graphitic type carbons formed from the pyrolysis-catalysis of waste tyre with different Al_2_O_3_ to SiO_2_ ratios (3:5, 1:1, 3:2 and 2:1) and different water injection rate (2 and 5 ml h^-1^).

The amounts of the different types of carbon produced on the catalysts are shown in Figure 5. The results show that as the Al_2_O_3_:SiO_2_ ratio was increased from 3:5 to 2:1, the filamentous carbon production increased marginally from 171.68 mg g^-1^ waste tyre to 179.12 mg g^-1^ at the ratio 1:1 and then decreased to 124.40 mg g^-1^.

#### Influences of different water injection rates

**Figure 6.**
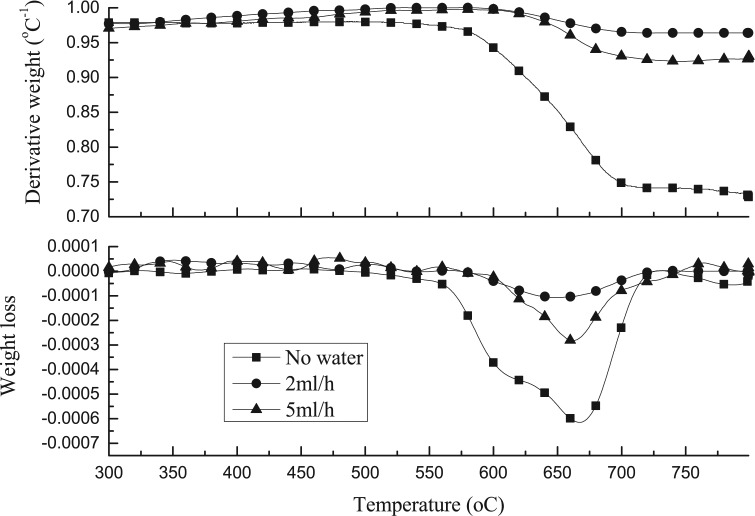
TPO and DTG-TPO results of used catalysts from pyrolysis-catalysis of waste tyres with 20 wt.% Al_2_O_3_/SiO_2_ catalyst at Al_2_O_3_ to SiO_2_ ratios of 1:1 and pyrolysis-catalytic steam reforming of tyres at different water injection rates (2 and 5 ml h^-1^).

**Figure 7.**
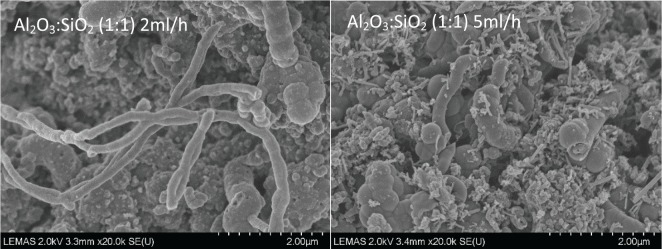
SEM images of carbon deposited on the catalysts from pyrolysis-catalysis of waste tyres with 20 wt.% Al_2_O_3_/SiO_2_ catalyst with Al_2_O_3_ to SiO_2_ ratio of 1:1 at different water injection rates (2 and 5 ml h^-1^).

**Figure 8.**
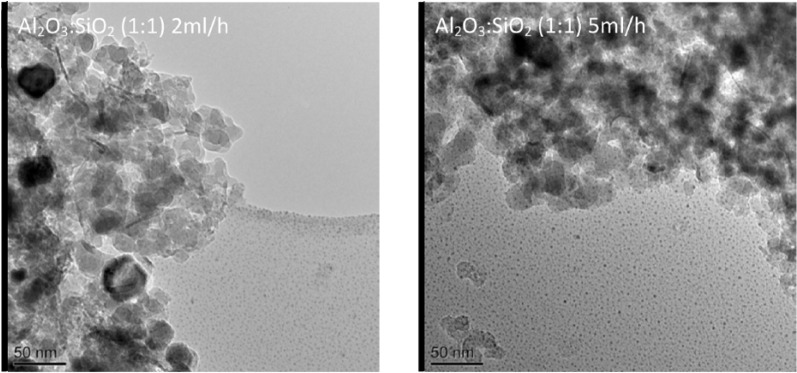
TEM images of carbon deposited on the catalysts from pyrolysis-catalytic reforming of waste tyres with 20 wt.% Al_2_O_3_/SiO_2_ catalyst with Al_2_O_3_ to SiO_2_ ratio of 1:1 and pyrolysis-catalytic steam reforming of tyres at different water injection rates (2 and 5 ml h^-1^).

Figure 6 shows the TPO and DTG-TPO results of the reacted catalysts from pyrolysis-catalytic steam reforming of waste tyres with the 20 wt.% Al_2_O_3_/SiO_2_ catalyst with an Al_2_O_3_ to SiO_2_ ratio of 1:1 at different water injection rates. The results show that the introduction of water leads to a significant decrease in the amount of carbon deposited on the catalyst. Filamentous carbons oxidized at high temperature were observed from the DTG-TPO results. SEM images in Figure 7 confirm filamentous carbon formation. Figure 8 shows the TEM analysis of the filamentous carbons formed with the 20 wt.% Al_2_O_3_/SiO_2_ catalyst (1:1 ratio) and different water injection rates, showing that they are mostly solid carbon fibres. Figure 4 showed earlier that for the 1:1 ratio Al_2_O_3_/SiO_2_ nickel catalyst in the absence of water (steam) input, the deposited carbons were MWCNTs. The Raman analysis of the carbons deposited on the catalysts in relation to input of steam (Table 2) shows that for the I_D_/I_G_ ratios for the deposited carbons for the experiments with 2 and 5 ml h^-1^ water injection rates are 0.87 and 0.90, which indicates the lower water injection rate contributes to higher graphitization of carbon formation.

Figure 3 shows that when water was introduced into the waste tyre pyrolysis-catalytic steam reforming process, less filamentous carbon was produced and less amorphous carbon was produced. As the water injection rate was increased from 0 to 5 ml h^-1^, the amount of filamentous carbon production decreased from 179.12 to 46.33 mg g^-1^ of waste tyre. Also, the amorphous carbon production decreased simultaneously. Therefore, the water introduction inhibited the carbon production in the waste tyre pyrolysis-catalytic reforming process and also inhibited filamentous carbon production.

The production of MWCNTs identified in this work for certain process conditions might have benefits to the process of hydrogen production instead of causing a problem to catalyst activity. There has been great interest in the application of carbon nanotubes in a wide variety of industrial sectors (Nessim, 2010; de Volder et al., 2013). Carbon nanotubes are currently produced from pure hydrocarbon feedstocks such as methane, benzene, acetylene, etc.; however, there is current interest in the use of waste materials such as plastics as alternative hydrocarbon feedstocks (Acomb et al., 2014; Arena et al., 2006; Chung and Jou, 2005; Liu et al., 2011; Zhang et al., 2008). Waste tyres are produced in large tonnages throughout the world and are usually collected as a separate waste stream, thereby aiding feedstock acquisition. The production of carbon nanotubes in the form of MWCNTs represents a potential valuable by-product from the production of hydrogen from waste tyres.

## Conclusions

In this paper, 20 wt.% Ni/Al_2_O_3_/SiO_2_ catalysts with four different Al_2_O_3_ to SiO_2_ mole ratios (3:5, 1:1, 3:2, 2:1) were studied for the pyrolysis-catalysis of waste tyres for hydrogen production and to determine the catalysts carbon deposition. The catalyst with an Al_2_O_3_ to SiO_2_ mole ratio of 1:1 gave the highest gas yield (27.3 wt.%), highest hydrogen production (14.0 mmol/g tyre) and highest syngas production (17.3 mmol/g tyre) when no steam was introduced to the system. In addition, the Ni catalysts with Al_2_O_3_ to SiO_2_ mole ratio 1:1 produced the highest deposition of carbon onto the catalyst at 164 mg g^-1^ of waste tyre. The carbon deposits were of the filamentous type and consisted of a large proportion of MWCNTs which have the potential to be of commercial value.

The introduction of steam to the process to produce pyrolysis-catalytic steam reforming of the waste tyres at different water injection rates was also investigated to determine the influence on both hydrogen and catalyst carbon deposition. The results showed that water addition to the process promoted higher hydrogen production and markedly reduced carbon deposition. The maximum yield of hydrogen produced was 34.5 mmol g^-1^_tyre_ at a water input rate of 5 ml h^-1^.As the water injection rate was increased from 0 to 5 ml h^-1^, the catalyst carbon deposition decreased from 19 to 4.6 wt.%.
